# Augmenting interferon-β and anti-PD-L1 antibody bolsters non-ablative radiotherapy to leverage potent abscopal effect in metastatic cancers

**DOI:** 10.3389/fimmu.2026.1925859

**Published:** 2026-09-01

**Authors:** Jin Hee Park, Hee Yeon Kim, Jihoon Choi, Hye Jung Kim, Ki Jung Ahn, Eun-tae Park, Kyun Do Byun, Jae Il Kim, Tae Hyun Kim, IL-Hwan Kim, SaeGwang Park

**Affiliations:** 1 Department of Microbiology, College of Medicine, Inje University, Busan, Republic of Korea; 2 Department of Surgery, Busan Paik Hospital, College of Medicine, Inje University, Busan, Republic of Korea; 3 Department of Radiation Oncology, Samsung Changwon Hospital, Sungkyunkwan University School of Medicine, Changwon, Republic of Korea; 4 Department of Radiation Oncology, Busan Paik Hospital, College of Medicine, Inje University, Busan, Republic of Korea; 5 Department of Surgery, Ilsan Paik Hospital, College of Medicine, Inje University, Goyang, Republic of Korea; 6 Department of Internal Medicine, Haeundae Paik Hospital, College of Medicine, Inje University, Busan, Republic of Korea; 7 NexThera Co., Ltd., Busan, Republic of Korea

**Keywords:** abscopal effect, immunotherapy, metastasis (cancer metastasis), radiotherapy, type - 1 interferons

## Abstract

**Background:**

The abscopal effect is a systemic anti-tumor immune response induced by local radiotherapy (RT), resulting in regression of distant, non-irradiated tumors. Although ablative RT is more effective than conventionally fractionated RT in inducing this response, the underlying mechanisms remain incompletely understood, and strategies to enhance the abscopal effect following conventional RT are still needed.

**Methods:**

We compared the immunological and therapeutic effects of ablative RT (15 Gy in a single fraction) and non-ablative RT (4 Gy in 10 fractions; biologically equivalent to 40 Gy) using murine breast cancer models. Tumor growth, immune cell infiltration, dendritic cell activation, cytokine expression and type I interferon signaling were analyzed. Functional studies were performed using CD8^+^ T cell depletion, IFNAR1 blockade, recombinant IFN-β administration and anti-PD-L1 treatment.

**Results:**

Ablative radiation induced abscopal response characterized by significant suppression of distant tumor growth, whereas non-ablative RT failed to elicit this effect despite comparable local tumor control. The abscopal response depended on CD8^+^ T cells and was associated with increased infiltration of activated CD8^+^ T cells into distant tumors. Ablative RT promoted the recruitment and activation of CD11c^+^ dendritic cells, particularly CD103_+_ cells, in both tumors and draining lymph nodes. The cytokine qPCR array suggested that increased signaling of type I IFN and the mRNA and protein levels of type I IFN, specifically IFN-β significantly upregulated in tumor tissues irradiated with ablative radiation. Blocking IFNAR1 abolished the abscopal response, whereas administration of recombinant IFN-β restored the ability of sub-ablative RT to induce systemic anti-tumor immunity. Furthermore, combining recombinant IFN-β with anti-PD-L1 therapy significantly enhanced the therapeutic efficacy of sub-ablative RT in murine breast, colon, and lung cancer models.

**Conclusions:**

Our findings identify IFN-β as a critical mediator of RT-induced abscopal immunity and demonstrate that IFN-β supplementation can overcome the limited systemic efficacy of conventional fractionated RT. These results provide a rationale for combining IFN-β with radiotherapy and immune checkpoint blockade to enhance systemic anti-tumor immunity.

## 1 Introduction

In contemporary oncology, approximately 60% of newly diagnosed cancer patients are scheduled to undergo radiotherapy (RT), either as a primary treatment modality or for palliative purposes (1). The conventional approach to external beam RT is effective in constraining localized tumor progression and involves the administration of multiple daily fractions of low-dose radiation energy over an extended span of several weeks. Noteworthy advancements in current radiation delivery apparatuses have ushered in an era marked by heightened potential to administer ablative radiation. Contemporary RT strategies have revealed a spectrum of immunomodulatory properties (2, 3).

One intriguing and infrequent phenomenon known as the “abscopal effect” has arisen wherein irradiation of a solitary tumor yields regression in distant, non-irradiated tumors. This phenomenon has manifested across diverse malignancies and has ignited profound interest in cancer immunotherapy as a prospective avenue for enhancing immune-mediated responses against cancer (4). Despite the long-standing awareness of the systemic repercussions of radiation exposure, documentation remains largely confined to sporadic case reports. Although the precise mechanisms and therapeutic approaches responsible for inducing the abscopal effect remain unclear, low-dose partial radiation therapy (such as 3 Gy with a cumulative dose of 30 Gy) presents formidable challenges in eliciting this effect and exerts a negligible influence on immune function (5–7). This phenomenon was predominantly observed in the context of immunogenic tumors, such as melanoma; however, its occurrence is on the rise owing to the advancement of immunotherapeutic modalities. The intricate underpinnings of the abscopal effect continue to elude comprehensive elucidation, yet it is theorized to hinge upon the activation of the immune system and the release of tumor-associated antigens (4, 8).

Tumor irradiation exerts a dual effect: obliteration of tumor cells and instigation of immune cascades via the release of tumor antigens and damage-associated molecular patterns. These events culminate in the maturation of dendritic cells, increased priming, and activation of cytotoxic T lymphocytes (CTLs). Moreover, tumor antigens act as proinflammatory mediators, thereby increasing the production of cytokines by monocytes. These cytokines (in concert with activated CTLs) orchestrate the obliteration of tumor cells within the irradiated locale and as they traverse metastatic foci, catalyzing tumor regression or eradication (9–11).

While the direct impact of radiation therapy on cancer cell growth via DNA damage is well-acknowledged, the seminal work of Vanpouille-Box et al. (12). posits that the presence of cytosolic DNA serves as a stimulus for the secretion of interferon-β (IFN-β) by cancer cells. This phenomenon occurs following the activation of the DNA sensor cyclic GMP-AMP synthase (cGAS) and its downstream effector stimulator of interferon genes (STING, TMEM173). Furthermore, Trex1 DNA exonuclease deficiency amplifies IFN-β production, thereby enhancing the potential for the abscopal effect after radiation therapy (13–15).

Although the abscopal effect remains infrequent, its burgeoning potential as a therapeutic paradigm has propelled heightened enthusiasm and extensive inquiries into the domain of cancer immunotherapy. The quest for a more profound comprehension of the fundamental mechanisms underlying the abscopal effect and the identification of discerning biomarkers capable of prognosticating their emergence is of paramount importance. Such advancements hold promise for the refinement of treatment strategies and enhancement of outcomes for a diverse spectrum of patients grappling with the daunting challenges of cancer.

This study described compelling evidence elucidating the induction of the abscopal effect in a TUBO tumor model. This effect was markedly pronounced following ablative radiotherapy (15 Gy administered in a single fraction) compared with sub-ablative radiotherapy (4 Gy administered over ten fractions). Our findings predicted the pivotal contributions of T cells and dendritic cells. Furthermore, we underscore the indispensable role played by IFN-β, as its depletion markedly diminishes the abscopal effect ensuing ablative radiotherapy. Moreover, we corroborate that the adjunctive administration of IFN-β augments the efficacy of sub-ablative radiotherapy in surmounting the impediments associated with abscopal effect induction. Furthermore, our investigations unveil the synergistic potency of IFN-β in conjunction with anti-PDL1 antibodies in augmenting the therapeutic efficacy of sub-ablative radiation across murine breast, colon, and lung metastatic cancer models.

## 2 Materials and methods

### 2.1 Cell culture

TUBO cells are a cloned cell line established *in vitro* from BALB/neuT mouse mammary carcinomas. The TUBO cell line used in this study was originally provided by Dr. Yang-Xin Fu (The University of Chicago, Chicago, IL, USA) and has been maintained in our laboratory since our previous report (16). CT-26 cells are a murine colorectal carcinoma cell line derived from BALB/c mice. TC-1 cells were derived from C57BL/6 lung epithelial cells. The TC-1 cell line was kindly provided by Prof. Tae Woo Kim (Korea University College of Medicine, Seoul, Republic of Korea). The CT26 murine colon carcinoma cell line was purchased from the American Type Culture Collection (ATCC, Manassas, VA, USA). TUBO cell lines were cultured *in vitro* in Dulbecco’s modified Eagle’s medium (DMEM), and CT-26 and TC-1 cell lines were cultured in Roswell Park Memorial Institute 1640 (RPMI-1640) medium. DMEM and RPMI-1640 were supplemented with 10% heat-inactivated fetal bovine serum (FBS) (Cytiva, Washington, D.C., USA), 10% NCTC-109 medium, 2 mM/L L-glutamine, 0.1 mM/L MEM nonessential amino acids, 100 units/mL penicillin and 100 µg/mL streptomycin. The cells were maintained at 37°C in a humidified incubator with 5% CO_2_ atmosphere.

### 2.2 Tumor challenge and treatment

Animal experiments were performed following the guidelines approved by the INJE University College of Medicine Institutional Animal Care and Use Committee (No.2021-030, 2022-004). All mice (6–8 weeks of age) used in this study were purchased from Orient Bio (Taejun, Korea). TUBO cells (1 × 10^6^ cells), CT-26 (1.5 × 10^5^ cells), and TC-1 (5 × 10^5^ cells) were inoculated subcutaneously into the back side of the head flank (irradiated local tumor) and tail flank (non-irradiated distant tumor). Tumor development was monitored daily, and the tumor size was measured twice per week. Tumor volumes were measured along three orthogonal axes (x, y, and z) and calculated as follows: tumor volume = (x × y × z)/2.

Tumor-bearing mice were locally irradiated to the head flank using a clinical linear accelerator system (CLINAC iX-S; Varian Medical Systems, Palo Alto, CA, USA). Mice were anesthetized with an intraperitoneal injection of ketamine (90 mg/kg) and xylazine (10 mg/kg) and then placed on a 1-cm-thick acrylic plate in the lateral decubitus position. To minimize damage to normal tissues, the mouse body was carefully placed outside the radiation field. The area outside the tumor tissue was covered with bolus radiation to maximize the radiation dose. The prescribed dose rate was 6 MV and 1 Gy at a dose rate of 600 MU/min with a source-to-surface distance of 100 cm. The irradiation field size was 20 × 4 cm, with a distance of 10 cm from the electron-scattering foil to the shield. The linear accelerator calibration used a TRS-398–6 MV photon beam. Thus, a single 15 Gy irradiation was selected as the optimal ablative dose in this animal model. The radiation dose was calculated based on the following results of the biologically effective dose (BED) calculation: 15 Gy 1 time (BED, 90.0) was ablative, while 4 Gy 10 times (BED, 93.3) was sub-ablative.

Depletion of CD4^+^ and CD8^+^ alpha T cells was achieved by intraperitoneal (i. p.) injection of GK1.5 or YTS 169.4 mAb (BioXcell, New Hampshire, USA) at 5 mg/kg on day 3 and 1 before irradiation, respectively. Depletion was evaluated by staining blood T cells two days after injection. We investigated the local irradiation ablative dosage for the tumors based on the selection criteria for radiation in this study. For the IFNAR-1 blockade (MAR1-5A3, BioXcell) experiment, mice were treated (7.5 mg/kg) 1 d before and after the radiation. Recombinant human IFN-β (Pepro Tech, Rocky Hill, NJ, USA) was treated 1 d after irradiation. Neutralizing PD-L1 (programmed death-ligand 1) was performed after irradiation on the day of i.p. administration at 5 mg/kg with 10F.9G2 (BioXcell).

### 2.3 Flow cytometry analysis

For local and distant tumor-infiltrating lymphocyte staining, cells were prepared from tumor-bearing mice 14 days after irradiation. The tumor tissue was placed in a 5 mL digestion solution (1.5 mg/mL collagenase IV (Roche, Basel, Switzerland), 0.4 mg/mL DNAse I (Sigma Aldrich, St. Louis, MO, USA) in RPMI-1640 medium) and chapped with scissors. After shaking for 20 min at 37°C, 0.05% ethylenediaminetetraacetic acid (EDTA) was added to stop the reaction. The suspension was passed through a 40 µm filter and washed two times with cold phosphate buffered saline (PBS). Cells were counted using trypan blue. To block the FC receptor, the cells were incubated with an anti-CD16/CD32 (ab93) antibody for 15 min. Thereafter, cells were stained with each antibody in the PBS: anti-CD45.2 (104), anti-CD3 (145-2C11), anti-CD4 (GK1.5), anti-CD8 (YTS156.7.7rMAb), anti-CD11b (M1/70), anti-CD11c (HL3), anti-CD70 (FR70), anti-CD80 (16-10A1), anti-CD86 (GL1), anti-CD103 (M290) and anti-MHCII (M5/114) for 15min at 25°C. All antibodies were purchased from BD Biosciences (San Jose, CA, USA) and samples were acquired with a Canto II cytometer and analyzed with Flow-Jo software (version 10.0.7).

### 2.4 Analysis of IFN-г production by CD8+ T cells

Tumor-specific interferon-gamma (IFN-γ) secretion was detected by ELISPOT assay kits (BD Biosciences). Polyvinylidine fluoride membrane-based 96 well plates were coated with anti-IFN-γ antibody and incubated overnight at 4°C. Tumor-bearing mouse splenocytes (2 × 10^5^ cells) were co-cultured with dendritic cells (DCs) that were fed overnight with tumor cell lysates prepared via repeated freezing and thawing (TUBO cells). Each experimental condition was performed in triplicate (three wells). The plates were imaged and evaluated using an ElSPOT reader (AID). Concanavalin A (conA) was used as a positive control for cell activation.

### 2.5 Immunohistochemistry and Immunofluorescence

Tumor tissues were fixed in 10% formalin overnight. Fixed tissues were processed into paraffin blocks and sectioned. Subsequently, the samples were deparaffinized and hydrated, and endogenous peroxidase activity was blocked. Antigen retrieval was performed using a heat-induced epitope retrieval (HIER) method. Slides were incubated in 0.01 M citrate buffer (pH 6.0) at 95 °C for 30 min in a water bath. After cooling to room temperature, the slides were rinsed with phosphate-buffered saline (PBS) for 5 min. The antibodies used for IHC staining were anti-CD8α (YTS156.7.7rMAb) and antibodies used for IF staining were anti-γH2AX (N1-431), anti-CD11c (HL3), and anti-STING (T3-680). Primary antibodies diluted 1:200 in blocking buffer were applied to the slides and incubated overnight at 4 °C. The slides were then washed with PBS, and the secondary antibody diluted 1:1000 in blocking buffer was added and incubated for 1 h at 25°C. Immunohistochemistry slides were incubated with 3,3’-diaminobenzidine (DAB) substrate for 5 min and counterstained with hematoxylin for 30 s. The slides were then dehydrated in graded ethanol (100, 90, 80, 70%), cleared in xylene, and mounted with a coverslip using a mounting medium. The slides were visualized under a bright-field microscope (NanoZoomer 2.0, Hamamatsu, Japan) and images were captured using a camera system. The nuclei of the slides for IF staining were stained with 4′,6-diamidino-2-phenylindole (DAPI) for 30 s and covered with a mounting medium. Images were acquired at 40 × magnification using a confocal fluorescence microscope (AX, Nikon, Tokyo, Japan) with the appropriate filters.

### 2.6 RNA preparation and quantitative RT-PCR

To determine the level of type I IFN in tumor cells, gene expression was analyzed using RT-PCR. mRNA was isolated using the TRI reagent (Thermo Fisher Scientific, Waltham, MA, USA). The extracted RNA was subjected to complementary cDNA synthesis using a TOPscript RT DryMIX Synthesis Kit (Enzynomics, Daejeon, Korea). The amplification efficiencies were determined using a dilution series of standard tumor samples. Quantification was performed using the efficiency-corrected ΔΔCq method. The relative quantities of the *Gapdh* reference gene were determined, and a normalization factor was calculated based on the geometric mean for internal normalization. The primers used in this study were as follows: UniqueAssay ID, qMmuCED0061481 for mouse IFN-α1, qMmuCED0050444 for mouse IFN-β1 and qMmuCED0027497 for mouse Gapdh (BioRad, Hercules, CA, USA). The PCR fidelity was determined by melting temperature analysis and visualizing the product on a 1.5% agarose gel.

### 2.7 Statistical analysis

Differences between groups were analyzed using an unpaired t-test. Error bars represent standard deviation. Data were analyzed using GraphPad Prism version 6 for Windows (GraphPad Software, San Diego, CA, USA). Unless otherwise specified, values of *p* < 0.05, 0.01, and 0.001 are denoted as *, **, and ***, respectively. Differences that were not statistically significant are left unnoted.

## 3 Results

### 3.1 15 Gy single irradiation significantly induced abscopal effect by suppressing primary and secondary tumor growth in the metastatic breast cancer model

This study aimed to evaluate the therapeutic efficacy of ablative and sub-ablative RT doses in primary and secondary tumors in murine breast cancer models. To establish a metastatic murine breast cancer model, primary tumor responses to 4 and 15 Gy irradiation doses were assessed to discern the superior dose for eliciting the abscopal effect in secondary tumors.

**Figure 1:**
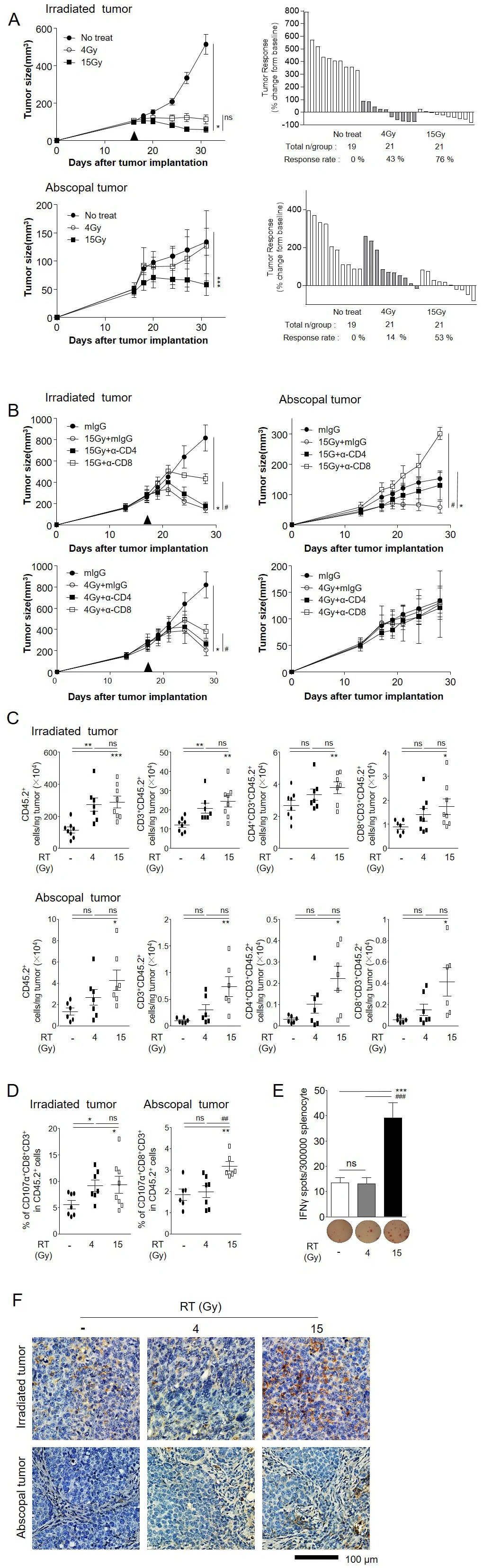
Regional irradiation with an ablative dose suppresses abscopal tumor growth through CD8⁺ T cells. **(A)** Tumors received a single 15-Gy dose or 4 Gy × 10 fractions over 2 weeks. Tumor growth was measured three times weekly. Black triangles indicate irradiation initiation. **(B)** CD4⁺ or CD8⁺ T cells were depleted by intraperitoneal injection of GK1.5 or YTS169.4 antibodies (5 mg/kg) 3 and 1 days before irradiation, respectively, and depletion was confirmed by flow cytometry. **(C–D)** Immune cell populations were analyzed by flow cytometry 7 days after irradiation. **(E)** Tumor antigen-specific responses were assessed by IFN-γ ELISPOT 14 days after irradiation. **(F)** Immune cell populations were analyzed by IHC 14 days after irradiation. Each group included 6–7 mice. All data represent the means ± SD, *P<0.05, **P<0.01, ***P<0.001, * is compared with the control group and # is compared with the radiation group.

Both 4 and 15 Gy irradiation doses demonstrated a remarkable capacity to markedly curtail primary tumor growth (**Figure 1A**). Ten days after irradiation, primary TUBO tumors in the 4 and 15 Gy irradiation groups exhibited a considerable reduction in size when juxtaposed with their sub-irradiated counterparts (*p* < 0.039). In contrast, secondary tumors displayed disparate responses depending on the administered radiation dose. Specifically, only the application of a 15 Gy irradiation dose successfully arrested the growth of secondary tumors, whereas the 4 Gy irradiation dose failed to do so (*p* < 0.001). The 4 Gy irradiation group achieved a therapeutic response rate of 43%, whereas the 15 Gy irradiation group achieved an impressively higher response rate of 76% in the context of primary tumors. Conversely, the 4 Gy irradiation group exhibited a therapeutic response rate of 14%, whereas the 15 Gy irradiation group attained a response rate of 53% in metastatic tumors (**Figure 1A**). Collectively, these findings unequivocally demonstrate that ablative radiation therapy employing a 15 Gy dose significantly engenders the abscopal effect within the metastatic TUBO model, whereas sub-ablative radiation therapy utilizing a 4 Gy dose failed to do so.

### 3.2 Ablative RT with 15 Gy induced an abscopal effect and recruited tumor-specific T cells in the secondary tumor

It is well known that the systemic immune response is the fundamental element of the abscopal response (2). To elucidate the immunological underpinnings of the abscopal effect, we conducted targeted CD4^+^ and CD8^+^ T-cell depletion experiments in a murine metastatic breast cancer model (**Figure 1**). CD8^+^ depletion experiments revealed a pronounced reliance on CD8^+^ T cells for the manifestation of abscopal responses. In mice subjected to CD8^+^ T cell depletion, the administration of a 15 Gy irradiation dose failed to induce abscopal responses in secondary tumors, in stark contrast to the robust abscopal tumor control observed in their non-depleted counterparts. Intriguingly, the 15 Gy irradiation regimen was associated with a reduction in primary tumor regression compared to the response observed in mice without CD8^+^ T cell depletion when CD8^+^ T cells were depleted (**Figure 1B**). Conversely, the 15 Gy irradiation dose consistently elicited abscopal tumor control, regardless of CD4^+^ T cell depletion. The administration of a 4 Gy irradiation dose did not engender an abscopal response, irrespective of T cell depletion status.

Tumor ablation recruits immune cells into the tumor and induces potent immunogenic cell death (ICD) by activating dendritic cell antigen presentation and CD8^+^ T cells (3). These activated CD8^+^ T cells then execute the primary treated tumor, shift into the periphery, and systemically distribute to target distant metastases as the driver of the abscopal effect (9). Seven days after local RT, exploration of the tumor-specific leukocyte population was performed to assess the initial impact of radiation on T cell activation within the primary tumor microenvironment. The CD3^+^CD45.2^+^ T cell population within primary tumors exhibited significant augmentation in response to 4 Gy and 15 Gy radiation treatment regimens. Subsequently, a comprehensive examination of the distinct T cell subsets was conducted within the tumor milieu. Corresponding disparities are observed in the composition of the T-cell population given the distinct therapeutic efficacies observed in secondary tumors following ablative and sub-ablative radiation.

In primary tumors, both radiation groups demonstrated robust recruitment of CD45.2^+^ and CD3^+^CD45.2^+^ T cells. In contrast, a significant augmentation in the populations of CD45.2^+^ and CD3^+^CD45.2^+^ T cells was exclusively observed in the 15 Gy irradiation group in sub-irradiated secondary tumors, modestly mirroring its therapeutic impact. Additionally, the accrual of CD4^+^ and CD8^+^ T cells was discernible within secondary tumors only after exposure to the 15 Gy irradiation regimen, consistent with the findings in primary tumors, with higher numbers observed in the 15 Gy group compared to the 4 Gy irradiated mice (**Figure 1C**).

The activation status of CD8^+^ T cells in the tumor was observed via an increase in the CD107a^+^ population in 4 Gy-and 15 Gy-irradiated primary tumors. In secondary tumors, an increase in the CD107a^+^ population was only observed in mice irradiated with 15 Gy (**Figure 1D**). Antigen-specific immune activity in the murine host was assessed using ELISPOT analysis. ELISPOT analysis conclusively showed the establishment of tumor-specific immunity during ablative irradiation (**Figure 1E**), as evidenced by a notable increase in IFN-γ spots observed in splenocytes following ablative radiotherapy. In contrast, sub-ablative radiation failed to induce a corresponding elevation in IFN-γ spots within the splenocytes.

Furthermore, the assessment of lymphocytic infiltration through immunohistochemical staining of tumors via tumor-infiltrating lymphocyte (TIL) scores revealed a more pronounced infiltration of CD8^+^ T cells in primary tumors subjected to 15 Gy irradiation (**Figure 1F**). This disparity in CD8^+^ T cell infiltration between the 15 Gy-irradiated and 4 Gy-irradiated groups extended to secondary tumors, where CD8^+^ T cell infiltration was exclusively evident in the sub-irradiated secondary tumors of the 15 Gy-irradiated mice, whereas it was absent in the 4 Gy-irradiated mice.

### 3.3 Ablative RT with 15 Gy primed DCs and the induced abscopal effect

Localized radiation in tumors prime DCs and re-energizes T cells (9). Tumor specimens and local lymph nodes were collected after local radiation to assess the potential disparities in systemic immunological responses triggered by ablative and sub-ablative radiation therapy, with a particular focus on assessing DC immune responses. The DC population and activation status within the tumors and lymph nodes were evaluated at 6, 24, and 48 h post-irradiation.

**Figure 2:**
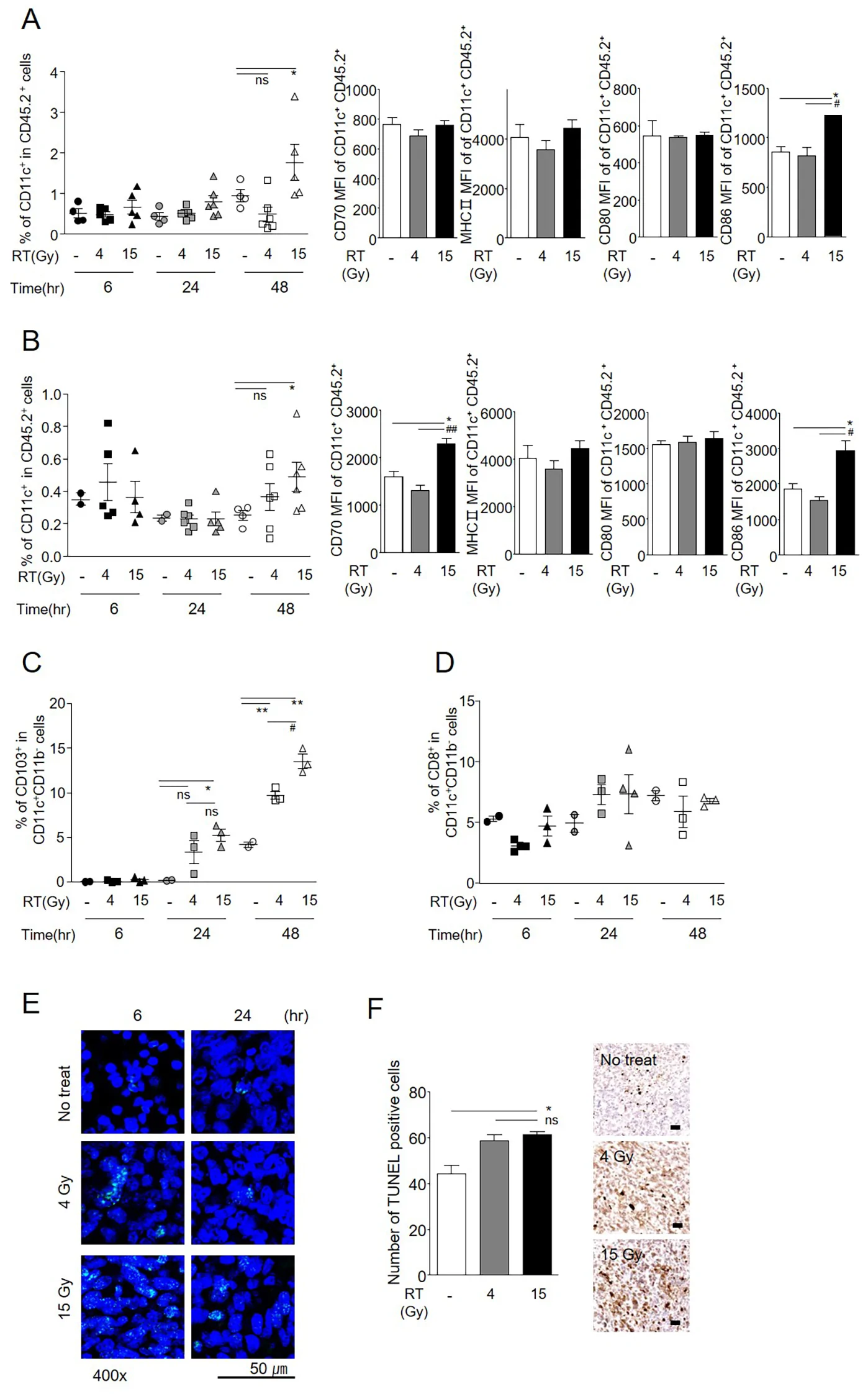
Ablative radiation induces an abscopal effect by increasing the migration and activity of DCs. **(A–B)** The distribution and activation of dendritic cells in irradiated tumors (A) and draining lymph nodes **(B)** were analyzed by flow cytometry (FACS) at 6, 24, and 48 h after irradiation with 0, 4, or 15 Gy. **(C–D)** Dendritic cell subsets were identified as CD11c⁺CD11b⁻CD103⁺ **(C)** and CD11c⁺CD11b⁻CD8α⁺ **(D)** cells in draining lymph nodes. **(E)** Radiation-induced DNA damage was assessed by immunofluorescence staining for γH2AX at 6 and 24 h after irradiation. **(F)** Radiation-induced cell death in tumors was evaluated using the TUNEL assay. Representative images were obtained at 400× magnification; scale bar, 50 μm. The number of mice analyzed in each experiment is indicated in the corresponding panels. All data are presented as means ± SD. P < 0.05, P < 0.01, and P < 0.001. * indicates a comparison with the control group, whereas # indicates a comparison with the radiation group.

There was a time-dependent increase in the proportion of CD11c^+^ cells within the CD45.2^+^ population in tumors following ablative radiation exposure (**Figure 2A**). Meanwhile, the DCs in the lymph nodes did not exhibit a time-dependent trend, although a notable augmentation in cell numbers and activation levels was discerned at the 48 h time point (**Figure 2B**). Additional analyses of DC subpopulations (especially CD103^+^ and CD8^+^ DC, which are mainly tumor-derived) were conducted. CD103^+^ DCs were significantly increased by sub-ablative and ablative RT 48 h after irradiation. However, ablative RT resulted in a noteworthy increase in CD103^+^ DCs compared to sub-ablative RT 48 h after irradiation. Only ablative RT evoked CD103^+^ DC increase in regional lymph nodes 24 hours after irradiation. In contrast, neither ablative nor sub-ablative RT activated CD8^+^ DC subpopulations in the regional lymph nodes (**Figures 2C, D**). In summary, the CD103^+^ subset of DCs that are recognized as being primarily tumor-derived showed a significant increase in their proportion after the use of ablative radiation doses.

To ascertain early-stage DNA damage following radiation exposure, immunofluorescence staining was employed to detect the expression of γH2AX in irradiated tumor cells. There was an increase in DNA damage within primary tumors following 4 and 15 Gy radiation treatments. Moreover, those subjected to 15 Gy radiation exhibited a higher degree of DNA damage than their 4 Gy-irradiated counterparts in the context of metastatic tumors (**Figure 2E**).

Apoptotic cell death within the tumor microenvironment was evaluated using a terminal deoxynucleotidyl transferase dUTP nick end labeling (TUNEL) assay. This study aimed to elucidate whether the distinct anti-tumor effects of ablative and sub-ablative radiation therapies are attributable to apoptotic or immunogenic responses. Remarkably, the TUNEL assay results following ablative and sub-ablative radiation treatments failed to establish any statistically significant differences, suggesting that the mechanism underlying the disparate systemic effects stemming from radiation dose variation may be more inclined toward immunogenic cell death rather than apoptosis (**Figure 2F**).

### 3.4 Chemokine gene expressions increased in the JAK-STAT pathway by ablative RT with 15 Gy

**Figure 3:**
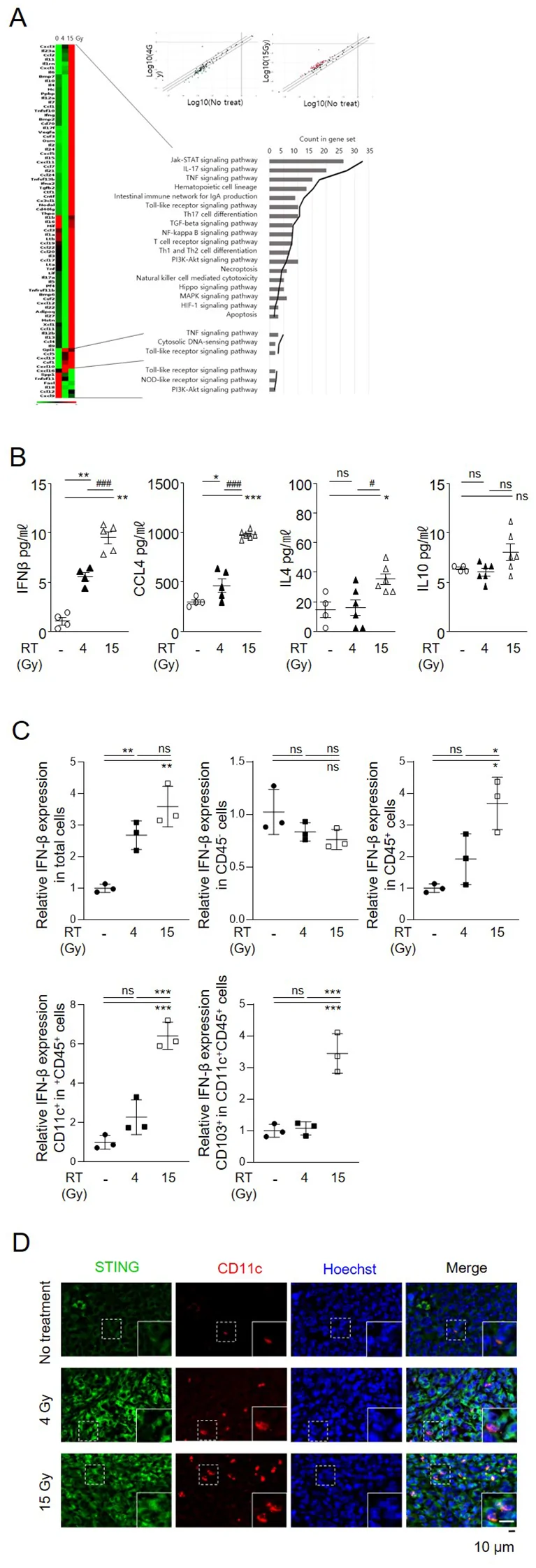
Ablative irradiation activates dendritic cells by increasing intratumoral cytokine and chemokine levels. **(A)** Cytokine and chemokine expression in irradiated tumor lysates was analyzed using an RT-qPCR array, with pathway enrichment analysis. **(B)** Cytokine levels, including IFN-β, CCL4, IL-4, and IL-10, were quantified after irradiation with different doses using multiplex analysis. **(C)** IFN-β expression in CD45⁻ and CD45⁺ tumor cells was assessed by RT-qPCR. CD11c⁺ and CD8⁺ cells were sorted from intratumoral immune cells, and type I IFN expression was analyzed by RT-qPCR. **(D)** STING protein expression in intratumoral CD11c⁺ cells was assessed by immunofluorescence staining. Representative images show STING (green), CD11c (red), and nuclei (blue). Scale bar, 10 μm. The number of mice analyzed is indicated in the corresponding panels. Data are representative of shown as individual values with mean ± SD, *P<0.05, **P<0.01, ***P<0.001.

Following the above experiment, we sought to clarify the main mechanism underlying the distinction in the systemic effects of local radiation between ablative and sub-ablative RT. Given the potential variations in radiation-induced by ablative and nonablative RT, we conducted a comprehensive cytokine and chemokine PCR array analysis to elucidate the repertoire of factors released within the tumor microenvironment. These factors encompass cytokines and chemokines emanating from immune and tumor cells. Immune cells release anti-inflammatory response factors that influence tumor cells. There was significant upregulation of genes associated with the JAK/STAT signaling pathway following ablative RT (**Figure 3A**). Furthermore, key components of the JAK/STAT signal transduction cascade (including CCL4, IL4, IL10, and IFN-β) exhibited notable increases at the protein level (**Figure 3B**).

To gain further insights, DCs (CD45^+^CD11c^+^) and T cells (CD45^+^CD8^+^) were isolated from intratumoral immune cell populations after radiation, and the expression of type I IFN genes was assessed using RT-qPCR. This investigation revealed a discernible augmentation of type I IFN (a hallmark of the JAK/STAT signaling pathway) predominantly in tumor tissues following ablative RT, with a particularly robust upregulation observed in CD11c^+^ cells. Among type I IFNs, IFN-β was more associated with systemic effects than IFN-α (**Supplementary Figure 1a**). Subsequently, we parsed the *IFN-β* gene expression in tumor tissue based on radiation dose and distinguished between CD45^-^ and CD45^+^ cells, confirming these distinctions through RT-qPCR. Intriguingly, CD45^+^ cells exhibited increased IFN-β expression solely in response to the ablative 15 Gy RT, whereas radiation treatment did not affect IFN-β expression in CD45^-^ cells. In particular, IFN-β gene was upregulated in DC cells by ablative 15 Gy RT, and was significantly increased in CD103^+^ DCs (**Figure 3C**).

Immunofluorescence assays corroborated these findings by demonstrating an increase in the STING signal within CD11c^+^ cells following ablative radiation, concomitant with elevated *type I IFN* gene expression (**Figure 3D**).

### 3.5 Anti-IFNAR-1 destroys the abscopal effect of ablative 15Gy RT

We devised a Type I IFN signaling-blocking antibody experiment to substantiate the indispensability of Type I IFN in the generation of the abscopal response following ablative RT.

**Figure 4:**
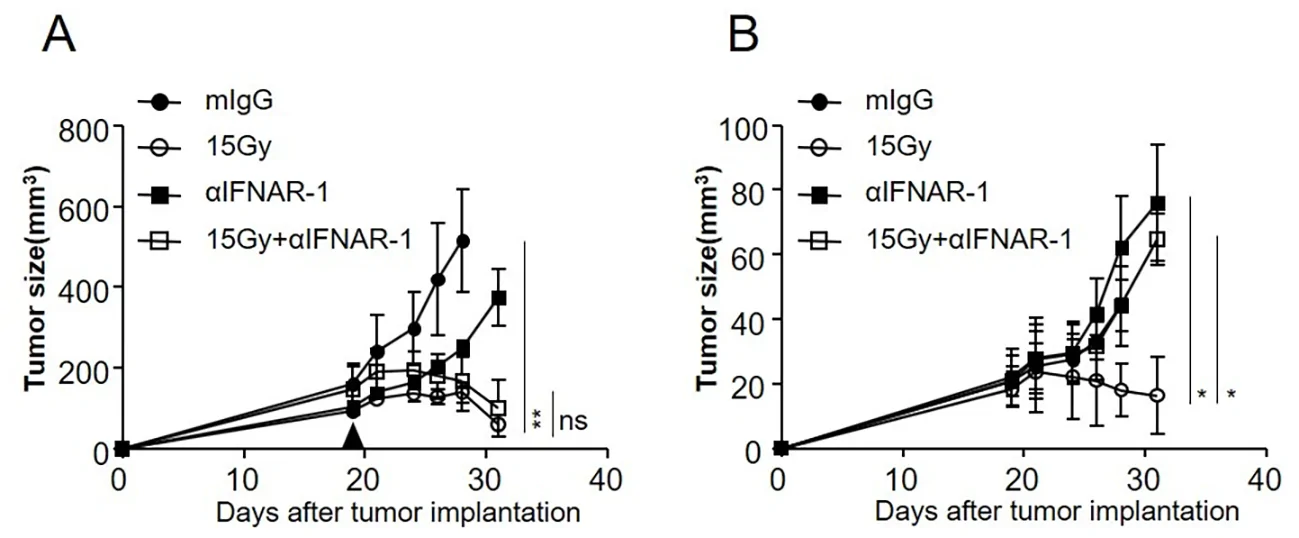
Type I IFN is increased by irradiation and is essential for inducing systemic antitumor effects. Type I IFN signaling was blocked by intraperitoneal injection of anti-IFNAR1 antibody (10 mg/kg) 2 days before and on the day of irradiation. (A) Irradiated tumor. (B) Abscopal tumor. Tumor growth was monitored after tumor implantation. Each experimental group consisted of six mice (n = 6). All data represent the means ± SD, *P<0.05, **P<0.01, * is compared with the 15 Gy radiation group.

The primary tumors regressed after ablative RT regardless of the anti-IFNAR-1 antibody injections (*p* < 0.003 and *p* < 0.01, respectively). Nonetheless, discernible variations were observed in the responses of secondary tumors within each experimental group. A reasonable abscopal response was induced after ablative RT of the primary tumor without the anti-IFNar-1 antibody, as evidenced by statistically significant inhibition of distant tumor growth (*p* < 0.027). In contrast, the therapeutic effect on distant tumors was markedly attenuated in mice subjected to anti-IFNar-1 antibody treatment (**Figure 4B**). Collectively, these findings underscore the essential role of type I IFN in eliciting an abscopal response to ablative RT.

### 3.6 Combination of an anti-PD-L1 agent and rh-IFN-β enhances the therapeutic efficacy of the abscopal effect by sub-ablative 4 Gy RT in the murine breast cancer model and in the murine colon and lung cancer models

The aforementioned experiments successfully demonstrated the occurrence of abscopal response induced by ablative RT, highlighting the limited therapeutic efficacy of sub-ablative low-dose RT, which is consistent with prior studies. However, the clinical application of ablative RT is limited by the associated hazardous side effects. Consequently, numerous studies have aimed to address these limitations and enhance the abscopal effects associated with sub-ablative RT. Building upon the insights gained from the above experiments which underscored the critical role of Type I IFN in generating the abscopal effect following ablative doses, we evaluated the sufficiency of Type I IFN in inducing an abscopal effect in 4 Gy sub-ablative RT (specifically IFN-β, which had increased expression in DCs) (**Figure 3**).

**Figure 5:**
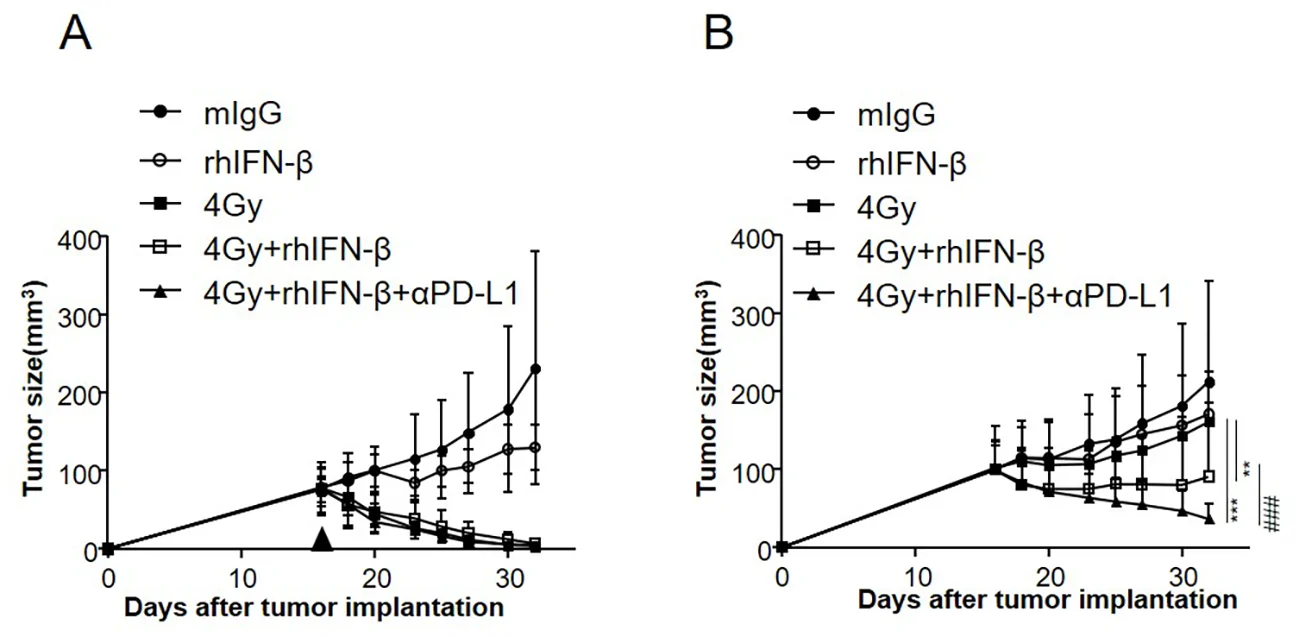
The addition of IFN-β induced an abscopal effect even following non-ablative irradiation. Recombinant human IFN-β (rhIFN-β) was intratumorally injected 48 h after irradiation, and PD-L1 was neutralized using an anti-PD-L1 antibody. (A) Irradiated tumor. (B) Abscopal tumor. Statistical significance of differences between controls and experimental groups were evaluated using t-test. *P < 0.05, **P < 0.01, and ***P < 0.001. * indicates a comparison with the 4Gy irradiation group, whereas # indicates a comparison with the 4Gy irradiation + rhIFN-β treatment group.

Capitalizing on the potential of IFN-β, we subjected mice to sub-ablative RT in the presence of recombinant human IFN-β (rh-IFN-β) and subsequently evaluated the growth of primary and secondary tumors. Encouragingly, IFN-β significantly potentiated the anti-tumor effect of sub-ablative RT in primary and secondary tumors (**Figures 5A, B**), thereby demonstrating its capability to enhance the potential of sub-ablative 4 Gy RT to elicit the abscopal response.

**Figure 6:**
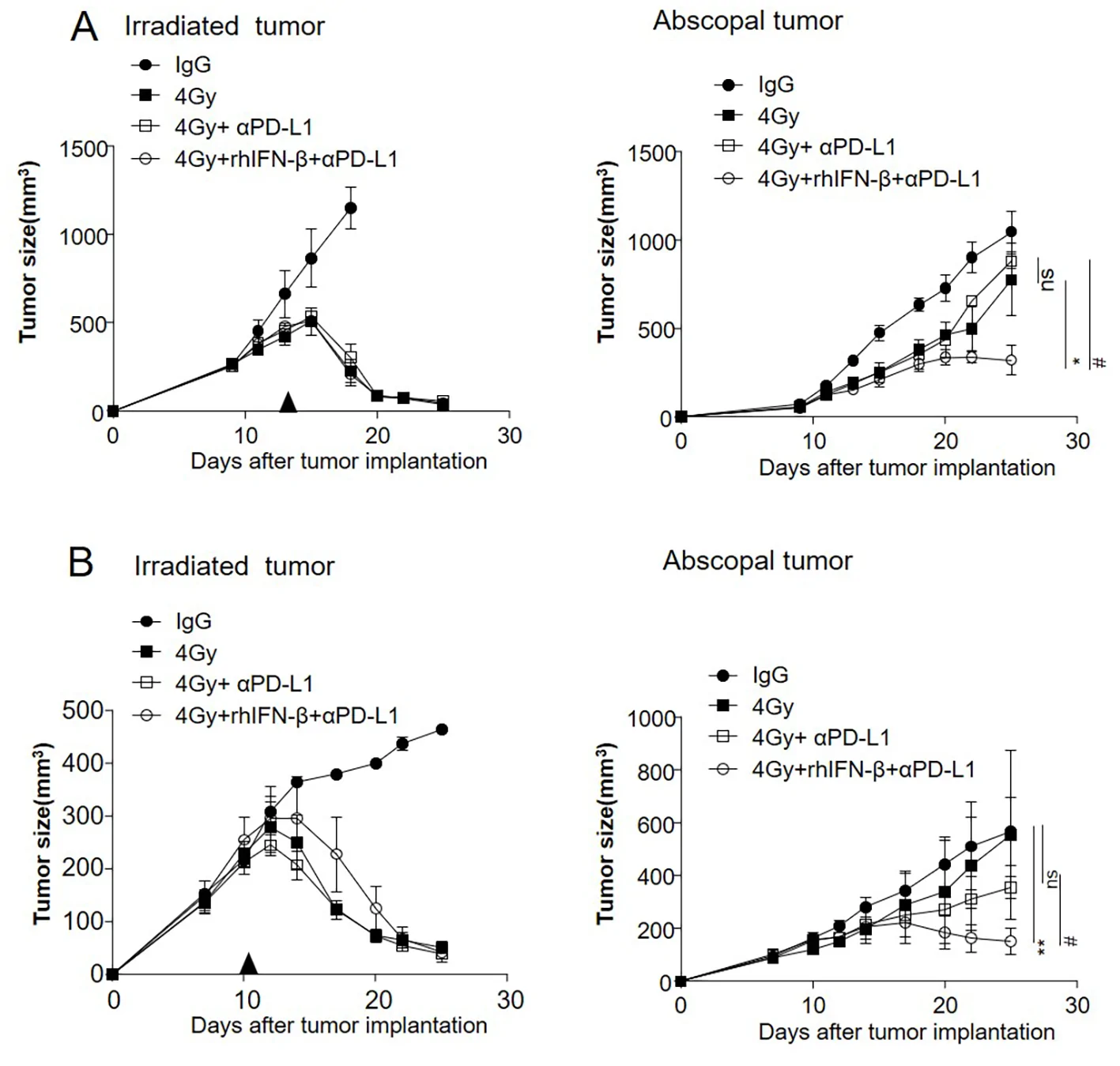
The combination of non-ablative irradiation with IFN-β and anti-PD-L1 enhanced systemic antitumor effects in multiple tumor models. **(A)** CT26 tumors. **(B)** TC-1 tumors. Irradiated and abscopal tumor growth was monitored after tumor implantation. Mice were treated with IgG, 4Gy irradiation, 4Gy irradiation + anti-PD-L1, or 4Gy irradiation + rhIFN-β + anti-PD-L1. Each experimental group consisted of six mice (n = 6). Statistical significance was evaluated using an unpaired two-tailed Student’s t-test. P < 0.05, P < 0.01, and P < 0.001. Asterisks (*) indicate comparisons with the 4-Gy irradiation group, whereas number signs (#) indicate comparisons with the 4-Gy irradiation + rhIFN-β + anti-PD-L1 group. ns, not significant.

Expanding our investigation to a metastatic mouse colon cancer model employing CT-26 cells, we applied sub-ablative RT to the primary tumor, followed by rh-IFN-β administration 48 hours later. Subsequent assessment of primary and secondary tumor growth in rh-IFN-β aided mice and IgG-injected mice consistently echoed the findings in the breast cancer model, with IFN-β effectively augmenting the anti-tumor effect of sub-ablative RT in primary and secondary tumors (*p* < 0.0003, and *p* < 0.005 respectively) (**Figure 6A**). Similarly, the combination of rh-IFN-β with sub-ablative RT successfully induced the abscopal effect in a mouse lung TC-1 cancer model (*p* < 0.002) (**Figure 6B**).

Given the well-established reliance on the abscopal effects on immunological mechanisms, it is plausible that cancer immunotherapy could synergize with RT to improve outcomes. Cancer immunotherapy has evolved into a well-established approach to enhance the efficacy of RT. Consequently, we introduced an anti-PD-L1 antibody to mice previously treated with rh-IFN-β to further amplify the clinical benefits of sub-ablative RT. Remarkably, this combination of anti-PD-L1 antibody and rh-IFN-β significantly augmented the abscopal effect induced by sub-ablative therapy across mouse breast TUBO, colorectal CT-26, and lung TC-1 cancer models (*p* < 0.001, *p* < 0.005, and *p* < 0.0002 respectively) (**Figures 5a–c**, respectively). These findings suggest the therapeutic potential of sub-ablative RT when aided with the combination of anti-PD-L1 antibody and rh-IFN-β for local tumor control and to achieve systemic control of breast cancer with reduced toxicity.

## 4 Discussion

Radiotherapy is a fundamental anti-cancer treatment. Its importance has increased when considering the palliation of a disease (1). Historically, the primary role of RT has been localized disease control. However, an expanding body of evidence concerning the immunomodulatory effects of RT has resulted in a paradigm shift in our understanding, shedding light on its potential to induce systemic anti-tumor responses. Among these notable phenomena, the abscopal effect is an archetypal example, wherein radiation targeting the primary tumor leads to the regression of distant metastatic lesions that remain non-irradiated. This intriguing effect has prompted investigations into the underlying immunogenic mechanisms. RT induces vascular inflammation (17) and primes antigen-presenting DCs (18), potentially playing a pivotal role in systemic responses. The main mechanism involves the activation of the cytoplasmic pattern recognition receptor STING via tumor-released DNA. This mechanism causes the crucial release of type I IFN and DC maturation (18). Radiation also induces immunogenic cell death to kill tumor cells (19). Harnessing this inherent systemic anti-tumor potential has long been promising in the field of systemic RT. Despite its initial conceptualization, the abscopal effect is rarely observed in real-world clinical settings, even in the face of extensive RT applications (20). Following robust studies to understand the precise mechanism of the abscopal effect to ultimately overcome this insufficiency of RT in the abscopal response, we propose the following schemes.

Determining the optimal dosage required to elicit an abscopal effect is challenging and often fraught with impracticality in clinical contexts. The efficacy of a systemic anticancer response significantly depends on the lethality inflicted upon tumor cells by RT. Irradiation plays a pivotal role in inducing lethal DNA double-strand breaks and instigating radiolysis within tumor cells, consequently generating reactive oxygen species that facilitate a cascade of DNA damage within the tumor microenvironment (21). Cumulatively, our findings align with those of previous research, indicating that ablation of primary tumors initiates a direct cytotoxic effect on tumor cells, subsequently reshaping the tumor microenvironment through the release of damage-associated chemokines and cytokines. This orchestration ultimately leads to the recruitment of immune cells to the tumor milieu, facilitates DC maturation, and activates CD8^+^ T cells, thereby instigating their assault on primary tumors and their dissemination to distant tumor sites. The proinflammatory cytokines integral to this immune activation process are released via the cyclic GMP–AMP synthase (cGAS)-stimulator of *IFN* genes (STING) pathway, and other inflammatory signaling cascades. The cGAS-STING pathway serves as a conduit for the production of interleukin-10 (IL-10). More importantly, IL-10 subsequently activates the JAK-STAT3 signaling which promotes the immunologic anti-tumor effect (22, 23). Taken together, ablative 15 Gy RT on the primary tumor exerted JAK-STAT activation, and we suggest that type I IFN is essential to activate the cGAS-STING pathway in mature DCs to lead to the systemic abscopal effect, which is concordant with previous literature (14).

Most recent studies revealed that the abscopal effect was only induced in fractionated moderate doses of radiation and not in doses that were too low or too high. An insufficient dose would not generate sufficient DNA damage to modulate the tumor microenvironment, but a superabundant dose might suppress anti-tumor immune responses. This eventually leads to a delicate therapeutic dose window for the abscopal effect, which generally ranges from 6 to <20 Gy per fraction (24, 25). Stronger RT leads to stronger cancer cell-intrinsic activation of the cGAS/STING pathway and type I IFN, leading to increased recruitment of DCs (26). Nonetheless, irradiation at doses exceeding 20 Gy can induce the expression of the exonuclease Trex1, which hinders the synergistic effects of RT by suppressing the activation of cytosolic DNA damage-sensing pathways. In the context of our study utilizing a primary murine breast cancer model, both sub-ablative 4 Gy and ablative 15 Gy radiation demonstrated radiosensitivity in terms of their impact on primary tumor control. However, only the ablative 15 Gy dose induced regression in distant secondary TUBO tumors. These findings imply that the disparity in abscopal responses is associated with the differential immune responses elicited by the two radiation doses. Initially, a higher radiation dose leads to the accumulation of double-stranded DNA (dsDNA) within the cytosol of primary cancer cells. Importantly, 15 Gy of radiation failed to generate an abscopal effect when CD8^+^ T cells were depleted. Additionally, the activation marker 107α in CD8^+^ T cells within the metastatic tumor model exhibited a significant increase following ablative 15 Gy RT. These results are consistent with the well-established theory that the immunological competence of patients plays a pivotal role in enhancing the efficacy of RT, ultimately determining the requisite radiation dose for effective tumor control (27). T-cells (particularly CD8^+^ T-cells) are required for the therapeutic effects of radiation. In a previous study on melanoma, deletion of CD8^+^ T cells dramatically weakened the therapeutic efficacy of radiation (9). Another study indicated that pre-existing intratumoral T cells (and not newly infiltrated T cells) govern the therapeutic effects of irradiation (28). Furthermore, ablative RT encompassed other significant changes in the tumor microenvironment that 4 Gy was incapable of causing. The cross-presentation of markers CD8, CD103, and CD86 in DCs increased. Furthermore, the IFN-γ of splenocytes and the DC population of regional lymph nodes increased after 15 Gy irradiation, which was representative of the systemic immune response leading to the abscopal effect. This modulation of the TME releases immune-stimulating tumor antigens increases T cell priming, and eventually systemically mediates immunogenic responses against distant tumors (29). This was consistent with the results of 15 Gy ablative irradiation in our study. The cGAS/STING signaling pathway was more prominent after ablative RT. Undoubtedly, the subsequent JAK/STAT-related cytokine expression was increased by 15 Gy of radiation. Our results suggest that ablative doses of 15 Gy of radiation are much more effective at inducing abscopal effects in breast cancer. Hence, it is clear why it is difficult to generate an abscopal effect in clinical settings (30). Assessing the safety profiles of therapeutic strategies is important to formulate an ideal treatment regimen, particularly given the challenges associated with the practical application of ablative doses in clinical settings. Although recent technological advancements have improved the precision of high-dose RT delivery to tumors, translation of these innovations into real-world medical practice remains a complex endeavor. While escalating the dose of RT may be considered to provoke an effective immune response when administered, the widely adopted regimen of 8 Gy delivered in three fractions was initially developed in murine model systems and has not been comprehensively validated in human clinical trials (31). Limited knowledge and expertise in elaborating a consistent system of high-dose radiation delivery in breast cancer trials across centers also provide a practical barrier to designing large multicenter trials. Thus, there is a demand for the expansion of standardized guidelines and dose constraints in the context of metastatic breast cancer and the abscopal effect.

To overcome the genuine dilemma of potential adverse effects of high-dose ablative RT, this study aimed to develop a breakthrough strategy to empower sub-ablative doses with the capacity to evoke the abscopal effect. Because sub-ablative RT was insufficient to induce abscopal RT, we focused on distinctive cytokines that demarcated 15 Gy from 4 Gy irradiation. Of note, the *IFN-β* mRNA level of DCs, and IFN-β of the tumor microenvironment were both elevated by ablative RT which successfully generated an abscopal response. After irradiation, damaged DNA fragments are discharged into the cytoplasm and engulfed by myeloid cells such as DC. This activates the cGAS/STING pathway and induces IFN-Is (especially IFN-β) IFN regulatory factor 3 (IRF3)/NK-κB-dependently (32, 33). IFNAR1 must be expressed by DCs to induce anti-tumor responses upon irradiation (34). This is consistent with the results of this study. When anti-IFNAR1 was treated, even 15 Gy of ablative RT lost its anti-tumor effect in the primary tumor and secondary tumor. In support of this, type 1 IFNs enhance radiosensitivity in cervical cancer (35, 36), glioma (37), non-small cell lung cancer (38), and pancreatic cancer (39), colorectal cancer (40). Radioresistant glioblastoma showed increased expression of IFN–JAK–STAT signaling regulators (SOCS1 and SOCS3) and attenuated IFN–JAK–STAT signaling (41). The importance of IFN-I signaling in determining RT efficacy has guided clinical endeavors to test combination therapies of RT and IFN-Is. Our study successfully provides the murine preclinical model in aiding ineffective sub-ablative RT with IFN-β to evoke an abscopal response. IFN-α was not significantly elevated like that of IFN-β when the 15 Gy irradiation generated an abscopal effect. Previous clinical trials on combination therapy of IFN-α and RT with a small group of melanoma patients cast doubts on their clinical benefits (42, 43). The dissimilarity between IFN-α and IFN-β should be further investigated.

Although recombinant human IFN-β-1a was used to evaluate the therapeutic potential of IFN-β augmentation, potential species-specific differences between human and murine type I interferon signaling should be considered. While the overall IFNAR signaling pathway is evolutionarily conserved, recent studies have demonstrated that human and murine IFN-β differ in receptor-binding properties and signaling characteristics, resulting in species-specific biological responses. Therefore, the therapeutic effects observed in this murine model should be interpreted with caution, and further validation using species-matched IFN-β or humanized IFNAR models will be important to facilitate clinical translation (44, 45).

Moreover, this study enhanced the effect of sub-ablative RT to induce an abscopal effect with IFN-β by adding an anti-PD-L1 immune checkpoint inhibitor. It is attractive to combine RT and immune checkpoint inhibitors to improve therapeutic efficacy owing to the immunogenic mechanism of radiation. Therefore, preclinical evidence on RT and ICB strategies is limited. Similar results were observed in our previous study using a murine hepatocellular carcinoma (HCC) model when only anti-PD-L1 was added to RT to boost the abscopal effect (46). However, the combination of IFN-β and anti-PD-L1 plus radiation to evoke the abscopal effect is an unsettled and novel field. Although rarely reported in clinical case reports, the abscopal effect of the triple combination of IFN, immunotherapy, and RT was introduced in renal cell carcinoma (47, 48) and melanoma (49). Nevertheless, none of them utilized IFN-β therapy. There is one ongoing clinical trial (NCT03474497) of a combination of cytokines, immunotherapy, and RT using IL-2. We warrant more future preclinical evidence with IFN-β and anticipate prospective studies to be held.

Recent approaches to administering RT generally utilize fractionation techniques involving multiple rounds or prolonged regimens. However, previous data suggests that the repeated regimens could induce adaptive immunosuppression via upregulation of PD-L1 and CD47 by yielding sustained IFN signaling in the tumor microenvironment (50) and production of immunosuppressive cytokine TGF-β (51). Therefore, rational strategies are to be confirmed to maximize immunostimulatory effects and minimize immunosuppressive effects, leading to the ultimate IFN-β+RT+ICB efficacy improvement. We urge the concluding specific regimen according to the tumor types, radiation dose, fractionation, types of RT (particles vs. photon), and the scheduling of IFN- β+RT+ICB. To our knowledge, this is the first attempt to utilize the combination of IFN-β and anti-PD-L1 therapy to enhance the sub-ablative RT to evoke the abscopal effect in murine breast, colon, and non-small cell lung cancer models.

The timing of immune checkpoint blockade relative to radiotherapy has emerged as an important determinant of therapeutic efficacy. Previous preclinical studies have demonstrated that concurrent or early administration of anti-PD-L1 antibodies during radiotherapy can produce superior antitumor responses compared with delayed sequential treatment, although the optimal schedule may vary depending on tumor type and radiation regimen. In the present study, we evaluated a single treatment schedule to investigate the immunological mechanisms underlying the abscopal effect rather than to optimize treatment sequencing. Future studies systematically comparing anti-PD-L1 administration before, during, and after ablative radiotherapy will be necessary to identify the most effective treatment schedule for clinical translation (52, 53).

Radiation dose and fractionation are critical determinants of the immunological consequences of radiotherapy. Emerging evidence suggests that different radiation regimens generate distinct patterns of DNA damage, inflammatory signaling, and immune cell recruitment, thereby shaping both innate and adaptive antitumor immune responses. Ablative radiation can induce robust cytosolic DNA accumulation and activate innate DNA sensing pathways, including the cGAS-STING axis, resulting in enhanced type I interferon production, dendritic cell activation, and CD8^+^ T-cell priming. In contrast, fractionated non-ablative radiation may elicit different DNA damage kinetics and immune regulatory programs, leading to distinct patterns of immune pathway activation (54). These findings suggest that the immunological outcome of radiotherapy is determined not only by the radiation dose itself but also by the fractionation schedule and the resulting dynamics of tumor–immune interactions. Our findings that ablative radiation preferentially enhanced IFN-β signaling and promoted the recruitment of CD103^+^ dendritic cells are consistent with this concept.

In the present study, ablative radiation induced stronger IFN-β expression and enhanced recruitment of CD103^+^ dendritic cells and CD8^+^ T cells compared with non-ablative radiation. These findings suggest that the magnitude and quality of radiation-induced immune activation, rather than radiation dose alone, determine the capacity of radiotherapy to induce systemic antitumor responses. Further investigation of dose-dependent regulation of immune signaling pathways will be important for optimizing immune-radiotherapy combinations.

Although STING expression was increased in CD11c^+^ cells following both 4 Gy and 15 Gy irradiation, IFN-β induction was preferentially enhanced after ablative radiation. STING functions as an upstream adaptor of the cGAS-STING pathway rather than a downstream effector of IFN-β signaling. Therefore, increased STING abundance alone may not directly reflect functional pathway activation. The differential IFN-β response between radiation regimens may be determined by additional regulatory events, including the extent of cytosolic DNA accumulation, cGAS activation, and downstream TBK1-IRF3 signaling. Furthermore, the absence of increased IFN-β signaling in CD45^-^ tumor cells suggests that radiation-induced type I interferon responses may predominantly occur within immune compartments, particularly dendritic cells, which are critical mediators of radiation-induced antitumor immunity.

Although T cells and host IFN-γ signaling are crucial for the abscopal effect by RT and ICBs (46), their exact mechanism has not been established to the best of our knowledge. The exact mechanism of action of the triple combination of type I IFN, immunotherapy, and RT remains unclear. We believe that this is the first attempt to utilize the combination of IFN-β and anti-PD-L1 therapy to enhance the sub-ablative RT to evoke the abscopal effect in murine breast, colon, and non-small cell lung cancer models. However, our results follow previous studies that emphasized the essential role of host IFNAR1 signaling in mediating RT and ICBs. Previous studies with either the use of IFNAR1 KO mice (15) or anti-IFNAR1 antibodies (55) completely deteriorated the synergistic effects of RT and Immuno-oncology drug (IO)s. Nonetheless, inadequate efforts (if any) are available to support the role of JAK-STAT in RT + IOs. Thus, although we have mechanistic knowledge of the role of the IFN–JAK–STAT axis in RT and IOs (56), more evidence is needed to clarify their involvement in various experimental settings.

## Data Availability

The datasets presented in this study can be found in online repositories. The names of the repository/repositories and accession number(s) can be found in the article/**Supplementary Material**.
